# Association Between Health Literacy and Frailty in Older Adults: A Study in Two Brazilian Municipalities

**DOI:** 10.3390/ijerph23070913

**Published:** 2026-07-16

**Authors:** Theda Manetta da Cunha Suter, Manoelito Ferreira Silva Junior, Marília Jesus Batista

**Affiliations:** 1 Department of Health, Centro Universitário Toledo Wyden, Araçatuba 16015-270, SP, Brazil; suter.theda@unitoledo.br; 2 Graduate Program in Health Sciences Faculdade de Medicina de Jundiaí, Jundiaí 13202-550, SP, Brazil; 3 Department of Health I, Universidade Estadual do Sudoeste da Bahia, Jequié 45208-409, BA, Brazil; manoelito.junior@uesb.edu.br; 4 Department of Public Health, Faculdade de Medicina de Jundiaí, Jundiaí 13202-550, SP, Brazil

**Keywords:** frailty, elderly people, health literacy

## Abstract

**Highlights:**

**Public health relevance—How does this work relate to a public health issue?**

- Frailty in older adults is a multifactorial syndrome associated with falls, hospitalizations, disability, and mortality, constituting a major public health problem in the context of an aging population.
- Health literacy, as a modifiable social determinant, directly influences autonomy and clinical outcomes, and may impact the prevention and management of frailty.

**Public health significance—Why is this work of significance to public health?**

- The study reveals a significant association between low health literacy and a higher risk of frailty, broadening our understanding of the social determinants that affect the elderly Brazilian population.
- This is one of the first studies in Brazil to highlight this relationship in older adults in the community, providing regional evidence to support public policies.

**Public health implications—What are the key implications or messages for practitioners, policy makers and/or researchers in public health?**

- Strategies aimed at enhancing health literacy should be incorporated into primary care as a means of preventing or reducing frailty in older adults.
- Public policies should consider the social, family, and clinical factors associated with frailty, promoting intersectoral actions for healthier aging.

**Abstract:**

Health literacy (HL), recognized as a modifiable social determinant, influences the autonomy and clinical outcomes of the elderly population, so that low levels of HL contribute to worse health outcomes, including frailty. The objective was to analyze the factors associated with frailty, with a focus on HL, in elderly people from two municipalities in the interior of São Paulo. In this cross-sectional study of 219 elderly individuals (≥60 years) from Jundiaí and Araçatuba, frailty was measured using the FRAIL-BR scale and the Clinical-Functional Vulnerability Index (IVCF-20), and HL was measured using the 14-item Health Literacy Scale (HLS-14). Sociodemographic, clinical, and behavioral variables were included. Bivariate analyses and multinomial logistic regression were performed, with a significance level of 5%. Women predominated (64.8%), with an average age of 71.8 (±7.55). Low HL remained associated with frailty according to the FRAIL scale (OR = 3.66; 95% CI: 1.51–8.85) and the IVCF-20 (OR = 5.65; 95% CI: 1.83–17.44). Other factors common to both scales were advanced age (FRAIL: OR = 4.24; 95% CI: 1.26–14.28; IVCF-20: OR = 28.67; 95% CI: 5.31–154.89) and family dysfunction (FRAIL: OR = 3.67; 95% CI: 1.06–12.68; IVCF-20: OR = 6.81; 95% CI: 1.72–27.06). Living alone and diabetes were associated with the IVCF-20, while depression was associated with the FRAIL scale. This research constitutes the first empirical study conducted in Brazil to provide evidence that low levels of health literacy are significantly associated with frailty in older adults.

## 1. Introduction

Health literacy (HL) has taken on a prominent role in the health management of frail older adults, emerging as an important factor in prevention and care [1] capable of mitigating frailty and acting in the transition to better outcomes [2,3].

HL may be regarded as the set of cognitive and social competencies that enable individuals to seek, understand, and apply information to promote and care for their own health. It unfolds in three dimensions: functional literacy refers to the basic reading and writing skills related to everyday information; communicative literacy involves the ability to interact and participate in health decisions; and critical literacy corresponds to the reflective analysis of information to exercise greater control over one’s own life [4].

Population aging is a complex, multidimensional phenomenon [5] and will soon be one of the most significant social transformations of this century [6]. Longevity represents an important victory, and the quest to ensure that these additional years are lived with quality is intense, especially in developing nations [7]. In Brazil, which is undergoing a rapid and intense demographic transition [8], the number of elderly people has grown by 57.4% in the last 12 years, and the aging index has risen from 30.7 to 55.2 in 2022 [9].

At the same time, health inequalities can be understood through the model of social determinants, organized into distal, intermediate, and proximal levels. Distal levels involve structural factors such as income and education; intermediate levels refer to living conditions, health literacy, and access to health services; and proximal levels relate to individual behaviors and conditions. These levels interact and contribute to vulnerability and frailty in older adults [10]. In relation to geriatric syndromes, frailty is evident as a relevant clinical condition which, although commonly related to aging, does not represent an inevitable consequence of this process [11].

Frailty in older adults is recognized as a multifactorial clinical syndrome characterized by a state of physiological vulnerability resulting from a decrease in energy reserves and an inability to maintain or recover homeostasis after stressful events. This condition results from a cumulative decline in multiple physiological systems, making the individual more susceptible to adverse outcomes such as falls, hospitalizations, disabilities, and mortality [12]. Since frailty is the cumulative result of stressful events experienced by older adults over the years, the hypothesis of our study is that health literacy is associated with health-related decision-making, which may or may not result in some degree of frailty, even when adjusted for socioeconomic factors.

Important research findings show that more vulnerable groups, especially older adults, have lower HL [13]. Despite this evidence, there is a gap in the scientific literature on the intersection between health literacy and frailty, especially in specific local contexts, such as the Brazilian population [14]. Studies addressing this relationship are particularly relevant, as they can provide valuable information to support public policies, given that this is a country with a high aging rate [15].

Understanding local dynamics is essential for developing effective interventions aimed at improving HL, which has been highlighted as a modifiable factor associated with frailty in older adults, as evidenced by studies linking low levels of education and health literacy to a higher prevalence of frailty in this population [16,17,18].

Thus, the objective of this study was to analyze the factors associated with frailty, with a focus on HL, in older adults residing in two municipalities in the interior of the state of São Paulo.

## 2. Materials and Methods

This is a cross-sectional and analytical study conducted in two cities in the interior of the state of São Paulo: Jundiaí and Araçatuba. The data were collected in Jundiaí between May 2023 and June 2024, and subsequently in Araçatuba between July 2024 and January 2025. The protocol, instruments, and outcome cutoffs were maintained throughout the study.

According to data from the Brazilian Institute of Geography and Statistics (IBGE) for 2022, the state of São Paulo had a population of 44,411,238, while the municipality of Jundiaí had an estimated population of 443,221 inhabitants, spread over a land area of 431.204 km^2^. The city has a healthcare network that includes seven hospitals and 35 Primary Health Care Units (PHCUs) distributed throughout the municipality, in addition to Family Health Units (FHUs), outpatient clinics, emergency rooms, and several other specialized centers [19].

In the same census, Araçatuba had a population of 200,124 inhabitants, spread over an area of 1,167,126 km^2^. It has five hospitals and 20 PHCUs [20].

In Jundiaí, the study was registered at the following Health Units: PHCU Fazenda Grande, PHCU São Camilo (FHU), PHCU Esplanada, PHCU Novo Horizonte Family Clinic, and at the Center for Community, Culture, Work and Income Generation.

In Araçatuba, assessments were carried out with elderly people from the Movimente-se Project at the Orlando Ramalho Sports Gymnasium, known as Matarazzo, and from the José Roberto Turrini, Jessy Villela dos Reis, Raimunda Souza Martinez, Alfredo Dantas de Souza, and Dr. Marco Aurelio Pereira PHCUs. The Movimente-se Project is a municipal project linked to the Raimunda Souza Martinez PHCU.

The PHCUs in Araçatuba and Jundiaí were selected to cover the different geographical regions of north, south, east, and west in each municipality. The choice took into account the recommendations of the municipal secretariats and the agreement of the units to participate in the study. Both the selection of the PHCUs and the elderly people was carried out on the basis of convenience.

People aged 60 years or older participated in this study. Individuals with stroke or Parkinson’s disease with moderate to severe functional impairment were not evaluated, as they presented limitations that could interfere with the administration and reliability of the self-report assessments (FRAIL-BR scale and structured questionnaires), compromising the consistency of the measures.

A sample calculation was performed based on the prevalence of frailty of 13.4% in the ELSI-Brazil study [21]. A significance level of 5% (α = 0.05), a deff of 2, and an error of 10% were adopted, resulting in a minimum of 108 elderly individuals. Anticipating a possible loss of 20%, the planned sample size was 135 elderly individuals to be approached in each municipality.

### 2.1. Data Collection

The assessments were conducted through face-to-face interviews at the PHCUs and at the participants’ residences, accompanied by a Community Health Agent, as well as at the Matarazzo Sports Gymnasium in Araçatuba.

All elderly individuals who were present at the location on the days when the assessments were conducted for data collection were approached to participate in the study during the collection period, until the sample size was complete. The assessments were conducted using a questionnaire and physical evaluation, and the variables are described below.

### 2.2. Covariates

The sociodemographic variables relating to distal health determinants were age (divided into ranges from 60 to 69, 70 to 79, and over 80 years), gender (male, female), and body mass index (BMI) (obese, overweight, up to normal weight). Due to the small number of underweight participants, this category was grouped with the normal weight category under “up to normal weight” in the statistical analyses.

The socioeconomic variables relating to living conditions and intermediate determinants of health were as follows: education (up to incomplete elementary school, complete elementary school, and high school and above), marital status (single, with partner), living arrangements (alone, with spouse, family member, or other); family functionality using the Family APGAR instrument [22] (good functionality, moderate or severe family dysfunction), family income (up to one minimum salary, two to three salaries, four or more), and health literacy (low, high), explained below as this is the explanatory variable of interest in the study.

The clinical variables, primary determinants, and health behaviors were as follows: frequency of visits to the PHCU (frequent, infrequent), hospitalization in the last year (yes, no), physical activity (sedentary (does not engage in any physical exercise), moderately active (more than 75 min of aerobic activity per week or strength training once or twice a week), and active (strength training three or more times a week or more than 150 min of moderate activity per week), medications (three or more per day, one to two medications per day, or no continuous medications); high blood pressure, diabetes, hypercholesterolemia (present, absent); Charlson comorbidity index [23] (moderate to very high and mild risk of death in the next year); functionality in Instrumental Activities of Daily Living (IADL) using the Lawton and Brody scale [24] (independent, partially or totally dependent); smoking and alcoholism (currently, formerly, never); depression using the Geriatric Depression Scale (GDS-15) [25] (normal, mild to moderate depression, severe depression), and cognition using the 10-Point Cognitive Screener (10-CS) [26] (normal, possible cognitive impairment and probable cognitive impairment); edentulism was considered as total tooth loss in both arches (yes or no) [27].

### 2.3. Explanatory Variable

Health literacy was assessed using the Brazilian version of the 14-item Health Literacy Scale (HLS-14) [28]. This instrument assesses literacy at the functional, critical, and communicative levels. It has 14 questions and answers on a Likert scale with scores ranging from 14 to 70 points. The cutoff point was identified, as in the validation article, based on the sample median, with a score of ≥42 considered high health literacy and a score of ≤41 considered low health literacy.

### 2.4. Outcome

The outcome of this study is frailty, measured by two instruments with a multidimensional approach: the FRAIL-BR scale [29] and the Clinical-Functional Vulnerability Index (IVCF-20) [30].

The FRAIL-BR frailty assessment tool consists of five simple self-reported questions that are easy to apply and were developed to screen for frailty syndrome in primary care. The questions are: “1—Do you feel tired? 2—Do you have difficulty climbing a flight of stairs? 3—Do you have difficulty walking a block? 4—Do you have more than 5 diseases (described by the instrument)? 5—Have you lost more than 5% of your weight in the last 6 months?” The score range is from 0 to 5 points, and 1 point is given for any affirmative answer. A score of 0 points is classified as robust, 1 to 2 points as pre-frail, and 3 to 5 points as frail.

The IVCF-20 was developed and validated in Brazil based on the Vulnerable Elders Survey-13 (VES-13) and the Tilburg Frailty Indicator (TFI), in addition to incorporating elements from other instruments widely used in the scientific literature. It is a multidimensional instrument with 20 assessment items covering eight dimensions considered predictors of functional decline and/or death in older adults, such as age, self-perceived health, instrumental and basic activities of daily living (ADL), cognition, mood, mobility, communication, and the presence of multiple comorbidities. The higher the assessment result, the greater the risk of clinical-functional vulnerability. Thus, those who score 0 to 6 points are classified as robust, those who score 7 to 14 points are classified as potentially frail, and those who score 15 points or more are classified as frail [30].

### 2.5. Statistical Analysis

The data were tabulated in Excel^®^ and transferred to Statistical Package for the Social Sciences (SPSS) version 20.0 (IBM Corp., Armonk, NY, USA). Pearson’s chi-square test was used to compare categorical variables in the bivariate analysis between the two outcomes and the independent variables. Variables with *p* < 0.20 were included in the multinomial logistic regression model, using the step-forward method. One model was conducted for frailty using the FRAIL-BR scale and another for the IVCF-20. A significance level of 5% (*p* < 0.05) was adopted. In the multinomial logistic regression analysis, the robust category was used as the reference category.

### 2.6. Ethical Aspects

This study was approved by the Research Ethics Committee under CAAE 63588022.0.0000.5412 for research in Jundiaí and CAAE 77617024.7.0000.5420 for research in Araçatuba. All participants who agreed to participate in the study signed the Free and Informed Consent Form in advance.

## 3. Results

In the municipality of Jundiaí, the sample consisted of 119 older adults, reaching the minimum calculated number. In Araçatuba, eight people were lost due to their refusal to provide data for the study and not meeting the minimum age requirement to participate. Even so, the minimum sample size was achieved, with 219 older adults participating in both municipalities. As shown in Table 1, the average age was 71.85 years (±7.55), the majority being female (64.8%, *n* = 142), white (63.9%, *n* = 140), with a predominant educational level of incomplete elementary school (42%, *n* = 92) and an income of up to one minimum wage (45.7%, *n* = 100). Most had stable family relationships (57.9%, *n* = 121) and reported not having a partner (52.5%, *n* = 115), but lived with a spouse, family member, or other person (74.1%, *n* = 160).

As for frequency of visits to the PHCU, the majority (72.6%, *n* = 159) go at least once a year and have not been hospitalized (73.5%, *n* = 161), have a sedentary lifestyle (52.1%, *n* = 114), and reported having good chewing ability during meals (64.6%, *n* = 117). Regarding the use of continuous medications, most (63.9%, *n* = 140) take three or more per day.

Among the participants, there was a high prevalence of chronic noncommunicable diseases such as hypertension (79%, *n* = 173) and diabetes (45.4%, *n* = 99), and in the city of Araçatuba, the rates were significantly higher than in Jundiaí for hypertension (*p* = 0.004) and hypercholesterolemia (*p* < 0.001). There was also a significant predominance of frail older adults (*p* = 0.012) in Araçatuba, as verified by the IVCF-20.

As for HL, a small majority (50.7%) had low literacy, and this condition was also significantly higher (*p* = 0.024) in Araçatuba.

Regarding the factors associated with frailty, according to the FRAIL scale, the total score for each variable was compared between the groups of frail, pre-frail and robust older adults, and it was observed (Table 2) that age, HL, physical activity, depression and cognition are strongly associated factors (*p* < 0.001). Other conditions are also involved: education (*p* = 0.021), family functionality (*p* = 0.024), frequency of visits to the PHCU (*p* = 0.003), diabetes (*p* = 0.021), alcoholism (*p* = 0.042), and comorbidities according to the Charlson index (*p* = 0.004).

The analysis of factors associated with frailty using univariate and multivariable logistic regression models demonstrated that low HL was associated with frailty when assessed by both the FRAIL-BR instrument (OR = 3.66; 95% CI: 1.51–8.85), as shown in Table 3, and the IVCF-20 (OR = 5.65; 95% CI: 1.83–17.44), with the corresponding results presented later in the manuscript.

When analyzing the same factors associated with frailty, in accordance with the IVCF-20 in the bivariate analysis, it can be observed (Table 4) that age, education, HL, number of continuous medications in use, depression, and cognition are significantly (*p* < 0.001) associated. In addition to these, marital status (*p* = 0.025), functionality (*p* = 0.03), family income (*p* = 0.030), physical activity (*p* = 0.003), diabetes (*p* = 0.016), and comorbidities according to the Charlson index (*p* = 0.001) are also associated with frailty.

It is observed that functional independence for performing Instrumental Activities of Daily Living (IADL), analyzed using the Lawton Scale, is significantly associated (*p* < 0.001) with frailty in both instruments. However, it is plausible to consider this a consequence of the condition of frailty, which is why it was not used in the regression model.

In the univariate model (Table 5) of factors associated with frailty, according to the IVCF-20 instrument, the following were observed: age (OR = 40.67; 95% CI: 9.67–171.04), family dysfunction (OR = 5.33; 95% CI: 2.12–13.41), no partner (OR = 2.90; 95% CI: 1.31–6.42), presence of comorbidities (OR = 6.19; 95% CI: 2.32–16.50), cognitive impairment (OR = 21.54; 95% CI: 5.23–88.78), diabetes (OR = 0.45; 95% CI: 0.21–0.95). Furthermore, regarding potential frailty, the following were observed: depression (OR = 14.40; 95% CI: 1.67–124.04), age (OR = 14.86; 95% CI: 4.04–54.59), presence of comorbidities (OR = 2.99; 95% CI: 1.18–7.55), diabetes (OR = 0.45; 95% CI: 0.25–0.83) (Table 5).

The results of the multivariate model adjustment indicate that, regardless of age, diabetes, living alone, and family functionality, HL is significantly associated with the probability of frailty (Table 5).

## 4. Discussion

In this study, HL has been associated with frailty, measured by both the FRAIL-BR scale and IVCF-20, and adjusted for socioeconomic factors, health behaviors, and clinical factors. It is recognized as an important social determinant of health by the World Health Organization (WHO) [31] and, in this respect, the present study is highly relevant as it is the first to empirically investigate this association in the Brazilian population [14].

This association has been studied in other countries in adults and the elderly, under the influence of other variables such as acculturation, social support [32], chronic kidney disease [18], heart failure [17], depression, high blood pressure, and diabetes [33]. It is clear that frailty is a complex syndrome that involves different domains and affects multiple physiological systems, in addition to being difficult to manage by patients, families, society, and public health authorities because it involves, in its origin, rigid factors that are less susceptible to change or intervention [34].

In this study, the median HL among participants is similar to the median among participants over 55 years of age in the validation article for the HLS-14 instrument used here [28]. Studies have shown that individuals with high levels of HL tend to adopt preventive behaviors and healthy lifestyles [35], capable of mitigating the risks of frailty [32]. This improved knowledge about health issues, motivation, and people’s skills in understanding, evaluating, and applying health information favors accurate decision-making, commitment to a balanced diet, regular physical activity, and adherence to prescribed treatments, in addition to contributing to the reduction of risk factors associated with chronic diseases and the promotion of general well-being [35]. It is important to emphasize that HL is a socioeconomic marker, highlighting the importance of social determinants of health in the context of frailty, regardless of the assessment tool used.

Along with low HL, advanced age and family dysfunction were common factors associated with frailty among the participants in this study, according to both instruments. In addition to these, living alone and having diabetes are clinical aspects associated with frailty that were identified by the IVCF-20 instrument. Furthermore, depression was associated with frailty, according to the FRAIL assessment.

This difference can be explained by the multidimensional approach of the IVCF-20, which encompasses functional, psychological, and social domains [25]. Independently of this, the FRAIL-BR scale, also used in this study, is widely used internationally and has proven to be a viable, time- and cost-effective instrument [28] for assessing frailty in older adults in community environments [36].

Associating HL with frailty in such a consistent manner is relevant for a fuller understanding of this social determinant that influences the health of the elderly population, since other clinical factors, which are already well established in the scientific literature, have also been associated with the frailty syndrome in older adults in Brazil. A longitudinal study by the SABE (Health, Well-Being and Aging) group conducted with 1399 elderly people for the purpose of describing the prevalence of frailty among the elderly, analyzed the associated factors and attributed the evolution of the syndrome frailty, as in the present study, to advanced age, lack of a partner, low socioeconomic and educational levels, the occurrence of multimorbidities, and cognitive impairment and limitations in functional capacity [37].

Other important factors such as depressive symptoms and diabetes were associated with frailty in this study, as well as in a study by Rede Fibra (Frailty in Elderly Brazilians), which aimed to investigate the influence of each item in determining frailty in 5532 community-dwelling older adults. In the same study, high blood pressure was also not significantly associated with the presence of frailty in older adults [38].

Aging is intrinsically linked to physiological changes that compromise homeostasis and increase vulnerability to adverse events. In addition, the presence of multiple chronic diseases (multimorbidity) contributes significantly to functional decline and loss of autonomy, which are central factors in the characterization of frailty [37]. However, the Charlson comorbidity index was not considered in the multivariate regression analysis due to conflict with the multimorbidity item contained in the IVCF-20 index, which is an outcome measure.

It should also be noted that family dysfunction, characterized by the family’s inability to offer support to the elderly, was also associated with frailty in this study through analysis with the two instruments used. Such an association has already been observed in another Brazilian cross-sectional study with 384 elderly people in Curitiba, Paraná, which strongly suggested that physical frailty among the individuals studied was related to the level of family dysfunction [39]. The same study highlighted the absence of research evaluating this association, demonstrating the need for such research.

Family dysfunction, by weakening emotional bonds and limiting social support for older adults, contributes to increased frailty. Frequent conflicts, inadequate communication, and lack of emotional support generate feelings of loneliness, anxiety, exhaustion, and low self-esteem, which directly impact physical and mental health. Reciprocal overload and stressful events between the elderly person and the caregiver, resulting from relational conflicts and the accumulation of physical and emotional demands, are closely associated with frailty and its progression. Evidence indicates that frailty is more prevalent in dysfunctional family contexts [40], confirming the findings of the present study.

While family dysfunction was associated with frailty, living alone was also associated with pre-frailty in this study. Living alone is an independent factor associated with frailty, as the absence of company and social interaction can trigger psychological repercussions, loneliness, and depression. In addition, there is a bidirectional relationship between depression and frailty, in which depression contributes to reduced physical activity and nutritional intake, and these factors aggravate frailty [41].

As for edentulism, the absence of all natural teeth, despite its association with frailty not being significant in the multinomial analysis of this study, was a variable that remained in the model for adjustment but was not significant in the end, only in the univariate analysis. It is a condition of older adults that should be interpreted as an important clinical marker of nutritional vulnerability because it severely compromises their chewing ability. Chewing quality was inversely related to frailty, suggesting that the chewing function is a determinant for maintaining nutritional well-being [42]. The prevalence of edentulism in older adults observed in the present study was 46.40% and was higher than the national average reported in the 2023 Brazilian Oral Health Survey, which was 36.48% [43].

Although the variable related to functionality, assessed using the Lawton Scale, was initially included in the analyses, its presence in the final model was discarded due to its significant impact in overestimating the odds ratio values and confidence intervals. However, the clinical relevance of this measure should be emphasized, since functional dependence is strongly associated with frailty [37]. It should be noted that, in this study, cognition and depression were included in the regression model because of the model adjustment and because they are conceptually important in the assessment of frailty. This is not a longitudinal study to infer risks, but it can be said that these aspects associated with frailty can be used as predictors and guide strategies for the prevention and reduction of harm.

Furthermore, it is important to highlight that although health literacy is positively associated with education, these constructs represent distinct dimensions. While educational attainment reflects formal education acquired over the course of a lifetime, health literacy encompasses the skills required to access, understand, evaluate, and apply health information in decision-making. In the present study, education was included as a covariate in the logistic regression model, and the association between low HL and frailty remained significant, suggesting the importance of health literacy on this outcome [44].

As for the limitations of this study, because it is a cross-sectional design and uses stepwise regression modeling, it is not possible to establish causal relationships or monitor the evolution of frailty over time based on the indicators observed. Thus, the results cannot be extrapolated. Longitudinal studies in other states are recommended.

Furthermore, the cities were chosen for the convenience of the researchers. Some characteristics of the sample differed between Jundiaí and Araçatuba, but in most variables the samples were similar, so the group of elderly people was combined for the regression analysis. It should also be noted that this study involved participants linked to the public health system, who are generally involved in health care services and demonstrate interest in promoting and improving their health conditions. The reported multivariable analyses, especially the more extreme OR estimates, should be interpreted with caution because of the small sample size. Additionally, HL was dichotomized using the sample-specific median of the HLS-14 score. Consequently, some degree of participant misclassification cannot be excluded, and the observed associations should be interpreted considering this methodological limitation.

The findings of this research provide support for the development of public policies and decision-making by health professionals in the early identification of individuals at risk of frailty, thereby enabling the implementation of preventive strategies aimed at health promotion and education before the outcome occurs. Moreover, although frailty represents a significant challenge for health systems, it is estimated that about 18% of individuals considered frail have the possibility of returning to a milder stage of the condition, such as pre-frailty, which demonstrates the importance of timely and targeted interventions to reverse or mitigate this clinical condition [45]. In the context of accelerated population aging in Brazil, the results of this study, which have identified HL as a factor associated with frailty, are particularly relevant. Understanding the multiple determinants of frailty, beyond the already widely recognized clinical aspects, allows for a broader perspective regarding the risks to which older adults are exposed [36].

Strategies such as including health topics in school curricula, simplifying the language used by health professionals, and adapting technical and scientific content to different levels of education and cultural adaptation are measures that can contribute to improving the health literacy of the population [46]. It is also important to emphasize the need to address these aspects in the training of health professionals, considering the role of health literacy as a modifiable factor to promote health.

## 5. Conclusions

It may be concluded that frailty is associated with HL, as measured by the FRAIL-BR scale and the IVCF-20. The present study broadens this scope by also identifying significant associations between frailty and two less explored aspects—low health literacy and family dysfunction—which contribute to a more comprehensive and multidimensional understanding of the phenomenon, in addition to age, depression, and living alone.

It is therefore recommended that health literacy be incorporated into future research on frailty, given its relevance as an associated factor, suggesting a multidimensional approach to this subject. Furthermore, its recognition as a strategic element in clinical management can contribute significantly to preventive and health promotion actions focusing on the elderly population.

## Figures and Tables

**Table 1:** Characteristics of older adults participating in the study as regards distal, intermediate, and proximal health determinants in the cities of Jundiaí, SP (*n* = 119) and Araçatuba, SP (*n* = 100), Brazil, 2025.

		Jundiaí *n* (%)	Araçatuba *n* (%)	Total *n* (%)	*p*
Distal Health Determinants (sociodemographic data)					
Age	80 to 99	18 (15.10)	20 (20.00)	38 (17.40)	0.610
	70 to 79	47 (39.50)	39 (39.00)	86 (39.30)	
	60 to 69	54 (45.40)	41 (41.00)	95 (43.40)	
Sex	Male	41 (34.50)	36 (36.00)	77 (35.20)	0.811
	Female	78 (65.50)	64 (64.00)	142 (64.80)	
Color/Race	White	84 (70.60)	56 (56.00)	140 (63.90)	**0.030**
	Black	34 (28.60)	39 (39.00)	73 (33.30)	
	Asian	1 (0.80)	5 (5.00)	6 (2.70)	
Body Mass Index	Obesity	29 (24.60)	19 (19.00)	48 (22.00)	0.085
	Overweight	37 (31.40)	46 (46.00)	83 (38.10)	
	Up to normal weight	52 (44.10)	35 (35.00)	87 (39.90)	
Intermediate Health Determinants (living conditions)					
Education	Up to incomplete elementary school	52 (43.70)	40 (40.00)	92 (42.00)	0.772
	EF	34 (28.60)	28 (28.00)	62 (28.30)	
	High school and above	33 (27.70)	32 (32.00)	65 (29.70)	
Marital Status	Without partner	61 (51.30)	54 (54.00)	115 (52.50)	0.686
	With partner	58 (48.70)	46 (46.00)	104 (47.50)	
Living With Whom	Alone	34 (29.30)	22 (22.00)	56 (25.90)	0.222
	Spouse, family member, or Other	82 (70.70)	78 (78.00)	160 (74.10)	
Family Functionality (APGAR)	Severe dysfunction	21 (19.30)	18 (18.00)	39 (18.70)	0.706
	Moderate dysfunction	23 (21.10)	26 (26.00)	49 (23.20)	
	Good functionality	65 (54.60)	56 (56.00)	121 (57.90)	
Family Income	Up to 1 MS	59 (49.60)	41 (41.00)	100 (45.70)	0.083
	2 to 3 MS	40 (33.60)	48 (48.00)	88 (40.20)	
	4 or more MS	20 (16.80)	11 (11.00)	31 (14.20)	
Health Literacy	Low	52 (43.70)	59 (59.00)	111 (50.70)	**0.024**
	High	67 (56.30)	41 (41.00)	108 (49.30)	
Proximal Health Determinants (clinical conditions and health behavior)					
Attendance at Neighborhood PHCUs	Does not attend	20 (16.80)	40 (40.00)	60 (27.40)	**<0.001**
	Yes, attends	99 (83.20)	60 (60.00)	159 (72.60)	
Hospitalized in the Last Year	Yes	19 (16.00)	38 (38.00)	57 (26.03)	**<0.001**
	No	100 (84.00)	62 (62.00)	162 (73.97)	
Physical Activity	Sedentary	70 (58.80)	44 (44.00)	114 (52.10)	**<0.001**
	Moderately active	26 (21.90)	12 (12.00)	38 (17.40)	
	Active	23 (19.30)	44 (44.00)	67 (30.60)	
Chewing While Eating	Poor	8 (9.90)	5 (5.00)	13 (7.20)	0.255
	Average	19 (23.50)	32 (32.00)	51 (28.20)	
	Good	54 (66.70)	63 (63.00)	117 (64.60)	
Medications Currently Taken	3 or more per day	76 (63.90)	64 (64.00)	140 (63.90)	0.571
	1 to 2 per day	36 (30.30)	33 (33.00)	69 (31.50)	
	None	7 (5.90)	3 (3.00)	10 (4.60)	
High Blood Pressure	Yes	85 (72.00)	88 (88.00)	173 (79.00)	**0.004**
	No	33 (28.00)	12 (12.00)	45 (20.60)	
Diabetes	Yes	57 (48.30)	42 (42.00)	99 (45.40)	0.351
	No	61 (51.70)	58 (58.00)	119 (54.60)	
Hypercholesterolemia	Yes	25 (21.20)	48 (48.00)	73 (33.50)	**<0.001**
	No	93 (78.80)	52 (52.00)	145 (66.50)	
Smoking	Currently	18 (15.10)	10 (10.00)	28 (12.80)	0.500
	Formerly	45 (37.80)	38 (38.00)	83 (37.90)	
	Never	56 (47.10)	52 (52.00)	108 (49.30)	
Alcoholism	Currently	13 (10.90)	14 (14.00)	27 (12.30)	0.657
	Formerly	22 (18.50)	21 (21.00)	43 (19.60)	
	Never	84 (70.60)	65 (65.00)	149 (68.04)	
Charlson Comorbidity	Moderate to very high	20 (16.80)	15 (15.00)	35 (16.00)	0.853
	Mild	99 (83.20)	85 (85.00)	184 (84.00)	
IADL (Lawton)	Partially dependent	15 (12.6)	20 (20.00)	35 (16.00)	0.097
	Independent	104 (87.4)	80 (80.00)	184 (84.00)	
Depression (GDS15)	Severe depression	6 (5.00)	2 (2.00)	8 (3.70)	0.199
	Mild depression	47 (39.50)	32 (32.00)	79 (36.10)	
	Normal	66 (55.50)	66 (66.00)	132 (60.30)	
Cognition (10-CS)	Probable impairment	8 (6.70)	8 (8.00)	16 (7.30)	0.824
	Possible impairment	35 (29.40)	26 (26.00)	61 (27.90)	
	Normal	76 (63.90)	66 (66.00)	142 (64.84)	
Vulnerability (IVCF-20)	Frail	13 (10.90)	26 (26.00)	39 (17.80)	**0.012**
	Potentially frail	45 (37.80)	28 (28.00)	73 (33.30)	
	Robust	61 (51.30)	46 (46.00)	107 (49.90)	
Frailty FRAIL-BR	Frail	25 (21.00)	28 (28.00)	53 (24.20)	0.360
	Pre-frail	54 (45.40)	37 (37.00)	91 (41.60)	
	Robust	40 (33.60)	35 (35.00)	75 (34.20)	
Edentulism	Yes	38 (46.30)	46 (46.50)	84 (46.40)	0.553
	No	44 (53.70)	53 (53.50)	97 (53.60)	
Total		119 (100)	100 (100)	219 (100)	

Source: Prepared by the authors, 2025. The significance value adopted was *p* < 0.05, chi-square test; significant values are highlighted in bold type. Note: Brazilian minimum salary = R$998.00 (June 2025). In some items, the percentage does not reach 100% because there was data loss in “Body Mass Index” (1), “Living With Whom” (3), “Family Functionality” (10), “Chewing While Eating” (38), “High Blood Pressure”, “Hypercholesterolemia” and “Diabetes” (1), and “Edentulism” (38). Legend: EF = Complete elementary school education, MS = Minimum salary, IADL = Instrumental Activities of Daily Living, PHCU = Primary Health Care Unit.

**Table 2:** Factors associated with frailty in older adults, according to the FRAIL-BR scale, SP, Brazil, 2025.

		FRAIL-BR				
	Variable	Robust *n* (%)	Pre-Frail *n* (%)	Frail *n* (%)	Total *n* (%)	*p*
Age	80 to 99	7 (9.30)	10 (11.00)	21 (38.50)	38 (17.00)	**<0.001**
	70 to 79	34 (45.30)	35 (38.50)	17 (32.70)	86 (39.40)	
	60 to 69	34 (45.30)	46 (50.50)	15 (28.80)	95 (43.60)	
Sex	Male	31 (41.30)	30 (33.00)	16 (28.80)	77 (34.90)	0.308
	Female	44 (58.70)	61 (67.00)	37 (71.20)	142 (65.10)	
Color/Race	White	44 (58.70)	64 (70.30)	32 (59.60)	140 (63.80)	0.538
	Black	29 (38.70)	25 (27.50)	19 (36.50)	73 (33.50)	
	Asian	2 (2.70)	2 (2.20)	2 (3.80)	6 (2.82)	
Body Mass Index	Obesity	11 (14.70)	23 (25.60)	14 (26.40)	48 (22.10)	0.107
	Overweight	33 (44.00)	36 (40.00)	14 (26.4.00)	82 (37.80)	
	Up to normal weight	31 (41.30)	31 (34.40)	25 (47.20)	87 (40.10)	
Education	Up to incomplete elementary school	24 (32.00)	38 (41.80)	30 (56.60)	92 (42.20)	**0.021**
	EF	20 (26.7)	28 (30.8)	14 (26.4.00)	62 (28.00)	
	High school and above	31 (41.30)	25 (27.50)	9 (17.00)	65 (29.80)	
Marital status	Single	35 (46.70)	50 (54.90)	30 (56.60)	115 (52.50)	0.449
	With partner	40 (53.30)	41 (45.10)	23 (43.40)	104 (47.50)	
Living With Whom	Alone	18 (24.00)	25 (28.10)	13 (25.00)	56 (25.90)	0.825
	Spouse, family member, or other	57 (76.00)	64 (71.90)	39 (75.00)	160 (74.10)	
Family Functionality (APGAR)	Severe dysfunction	8 (11.30)	16 (18.40)	15 (29.40)	39 (18.70)	**0.024**
	Moderate dysfunction	13 (18.30)	21 (24.10)	15 (29.40)	49 (23.40)	
	Good functionality	50 (70.40)	50 (57.50)	21 (41.20)	121 (57.90)	
Family Income	Up to 1 MS	27 (36.00)	42 (46.20)	31 (58.50)	100 (45.70)	0.147
	2 to 3 MS	34 (45.30)	37 (40.70)	17 (32.10)	88 (40.20)	
	4 or more MS	14 (18.70)	12 (13.20)	5 (9.40)	31 (14.20)	
Health Literacy	Low	25 (33.30)	44 (48.40)	39 (73.60)	108 (49.30)	**<0.001**
	High	50 (66.70)	47 (51.60)	14 (26.40)	111 (50.70)	
Attendance at Neighborhood PHCU	No	26 (34.70)	29 (31.90)	5 (9.40)	60 (27.40)	**0.003**
	Yes	49 (65.30)	62 (68.10)	48 (90.60)	159 (72.60)	
Physical Activity	Sedentary	21 (28.00)	52 (57.10)	41 (77.40)	114 (52.00)	**<0.0001**
	Moderately active	17 (22.70)	15 (16.50)	6 (11.30)	38 (17.40)	
	Active	37 (49.30)	24 (26.40)	6 (11.30)	67 (30.60)	
Chewing While Eating	Poor	5 (7.20)	4 (5.80)	4 (9.30)	13 (7.20)	0.088
	Average	12 (17.40)	22 (31.90)	17 (39.50)	51 (28.20)	
	Good	52 (75.40)	43 (62.30)	22 (51.20)	117 (64.60)	
Medications Currently Taken	3 or more per day	39 (52.00)	58 (63.70)	43 (81.10)	140 (63.90)	**0.013**
	1 to 2 per day	31 (41.30)	29 (31.90)	9 (17.0)	69 (31.50)	
	None	5 (6.70)	4 (4.40)	1 (1.90)	10 (4.60)	
High Blood Pressure	Yes	59 (78.70)	74 (81.30)	40 (76.90)	173 (79.40)	0.809
	No	16 (21.30)	17 (18.70)	12 (23.10)	45 (20.60)	
Diabetes	Yes	25 (33.30)	50 (54.90)	24 (46.20)	99 (45.90)	**0.021**
	No	50 (66.70)	41 (45.10)	28 (53.80)	119 (54.60)	
Hypercholesterolemia	Yes	25 (33.30)	34 (37.40)	14 (26.90)	73 (33.50)	0.445
	No	50 (66.70)	57 (62.60)	38 (71.10)	145 (66.50)	
Alcoholism	Never	50 (66.70)	70 (76.90)	29 (54.70)	149 (68.10)	**0.042**
	Formerly	14 (18.70)	12 (13.20)	17 (32.10)	43 (19.60)	
	Currently	11 (14.70)	9 (9.90)	7 (13.20)	27 (12.30)	
Charlson Comorbidity Index	Moderate to very high	5 (6.70)	15 (16.50)	15 (28.30)	35 (16.00)	**0.004**
	Mild	70 (93.3)	76 (83.50)	38 (71.70)	184 (84.00)	
IADL (Lawton)	Partially dependent	2 (2.70)	13 (14.30)	20 (37.70)	35 (16.00)	**<0.0001**
	Independent	73 (97.30)	78 (85.70)	33 (62.30)	184 (84.00)	
Depression (EGD15)	Serious depression	1 (1.30)	3 (3.30)	4 (7.50)	8 (3.70)	**<0.0001**
	Mild depression	14 (18.70)	33 (36.30)	32 (60.40)	79 (36.10)	
	Normal	60 (80.00)	55 (60.40)	17 (32.10)	132 (60.30)	
Cognition (10CS)	Probable impairment	2 (2.70)	5 (5.50)	9 (17.00)	16 (7.30)	**<0.0001**
	Possible impairment	16 (21.30)	23 (25.30)	22 (41.50)	61 (27.90)	
	Normal	57 (76.00)	63 (69.20)	22 (41.50)	142 (64.80)	
Edentulism	Yes	25 (36.20)	31 (45.60)	28 (63.60)	84 (46.40)	**0.017**
	No	44 (63.80)	37 (54.40)	16 (36.40)	97 (53.60)	
Total		75 (100)	91 (100)	53 (100)	219 (100)	

Source: Prepared by the authors, 2025. The significance value adopted was *p* < 0.05, chi-square test; significant values are highlighted in bold type. Note: Brazilian minimum salary (MS) = BRL 1518.00 (June 2025). In some items, the percentage does not reach 100% because there was data loss in “Body Mass Index” (1), “Living With Whom” (3), “Family Functionality” (10), “Chewing While Eating” (38), “High Blood Pressure”, “Hypercholesterolemia” and “Diabetes” (1), and “Edentulism” (38). Legend: EF = Complete elementary school education, MS = Minimum salary, IADL = Instrumental Activities of Daily Living, PHCU = Primary Health Care Unit.

**Table 3:** Factors associated with pre-frailty and frailty in older adults using logistic regression analysis in accordance with the FRAIL-BR scale, São Paulo, Brazil, 2025.

		Pre-Frail and Frail (Versus Robust)					
	Variable	Univariate Model			Multiple Model		
		Crude OR	CI 95%	*p*	Adjusted OR	CI 95%	*p*
	Health Literacy						
Pre-frail	Low	1.87	0.99–3.52	0.052	1.87	0.93–3.75	0.080
	High	1			1		
Frail	Low	5.57	2.56–12.11	**<0.001**	3.66	1.51–8.85	**0.004**
	High	1			1		
	Sex						
Pre-frail	Male	0.70	0.37–1.32	0.267			
	Female	1					
Frail	Male	0.61	0.29–1.29	0.199			
	Female	1					
	Education						
Pre-frail	Up to incomplete elementary school	1.96	0.942–4.09	0.072			
	EF	1.74	0.80–3.78	0.165			
	High school and above	1					
Frail	Up to incomplete elementary school	4.31	1.72–10.76	**0.002**			
	EF	2.41	0.88–6.61	0.087			
	High school and above	1					
	Marital Status						
Pre-frail	Single	1.39	0.75–2.57	0.289			
	With partner	1					
Frail	Single	1.49	0.73–3.02	0.269			
	With partner	1					
	Age						
Pre-frail	80 to 99	1.06	0.36–3.06	0.920	0.69	0.22–2.18	0.259
	70 to 79	0.76	0.40–1.45	0.408	0.64	0.31–1.29	0.209
	60 to 69	1			1		
Frail	80 to 99	6.80	2.38–19.42	**<0.001**	4.24	1.26–14.28	**0.020**
	70 to 79	1.13	0.49–2.63	0.771	1.01	0.38–2.63	0.991
	60 to 69	1			1		
	Living With Whom						
Pre-frail	Alone	1.24	0.61–2.50	0.553	1.25	0.56–2.75	0.584
	Spouse, family member or other	1			1		
Frail	Alone	1.06	0.46–2.4	0.897	0.76	0.26–2.21	0.621
	Spouse, family member or other	1			1		
	Family Income						
Pre-frail	Up to 1 MS	1.81	0.73–4.51	0.199			
	2 to 3 MS	1.27	0.52–3.12	0.603			
	4 MS or more	1					
Frail	Up to 1 MS	3.21	1.02–10.09	**0.045**			
	2 o 3 MS	1.4	0.43–4.53	0.575			
	4 MS or more	1					
	Alcoholism						
Pre-frail	Currently	1.71	0.66–4.44	0.269			
	Formerly	1.05	0.32–3.38	0.938			
	Never	1					
Frail	Currently	0.91	0.32–2.61	0.863			
	Formerly	1.91	0.58–6.23	0.284			
	Never	1					
	Medications						
Pre-frail	3 or more medications per day	0.54	0.14–2.13	0.377			
	1 to 2 medications per day	0.63	0.33–1.20	0.161			
	No continuous medications	1					
Frail	3 or more medications per day	0.18	0.02–1.62	0.127			
	1 to 2 medications per day	0.263	0.11–0.62	**0.002**			
	No continuous medications	1					
	Diabetes						
Pre-frail	Diabetic	2.44	1.30–4.59	**0.006**			
	No	1					
Frail	Diabetic	1.71	0.83–3.55	**0.146**			
	No	1					
	Body Mass Index						
Pre-frail	Obesity	2.09	0.87–5.01	**0.098**			
	Overweight	1.09	0.55–2.17	0.804			
	Up to normal weight	1					
Frail	Obesity	1.58	0.61–4.08	0.346			
	Overweight	0.53	0.23–1.19	0.124			
	Up to normal weight	1					
	Attendance at Neighborhood PHCU						
Pre-frail	No	0.88	0.46–1.69	0.703			
	Yes	1					
Frail	No	0.20	0.07–0.55	**0.002**			
	Yes	1					
	Charlson Comorbidity Index						
Pre-frail	Moderate to very high risk	2.76	0.95–8.00	0.061			
	Slight risk	1					
Frail	Moderate to very high risk	5.68	1.91–16.84	**0.002**			
	Slight risk	1					
	Family APGAR						
Pre-frail	Severe family dysfunction	2.00	0.78–5.09	0.146	1.44	0.52–3.98	0.480
	Moderate family dysfunction	1.615	0.73–3.58	0.237	1.35	0.59–3.09	0.477
	Good family functionality	1			1		
Frail	Severe family dysfunction	4.46	1.65–12.11	**0.003**	3.67	1.06–12.68	**0.040**
	Moderate family dysfunction	2.75	1.12–6.76	**0.028**	2.55	0.87–7.49	0.088
	Good family functionality	1			1		
	Depression (EGD15)						
Pre-frail	Serious depression	3.27	0.33–32.40	0.311	2.41	0.23–25.52	0.465
	Mild to moderate depression	2.57	1.25–5.31	**0.011**	2.14	0.94–4.88	0.071
	Normal	1			1		
Frail	Serious depression	14.11	1.48–134.82	**0.021**	6.92	0.65–73.54	0.109
	Mild to moderate depression	8.07	3.53–18.45	**<0.001**	3.40	1.31–8.85	**0.012**
	Normal	1			1		
	Instrumental Activities of Daily Living (Lawton)						
Pre-frail	Partially dependent	6.08	1.33–27.88	**0.020**			
	Independent	1					
Frail	Partially dependent	22.12	4.88–100.19	**<0.001**			
	Independent	1					
	Cognition						
Pre-frail	Probable impairment	2.26	0.42–12.12	0.341			
	Possible impairment	1.30	0.63–2.70	0.481			
	Normal	1					
Frail	Probable impairment	11.66	2.33–58.28	**0.003**			
	Possible impairment	3.56	1.58–8.01	**0.002**			
	Normal	1					
	Physical Activity						
Pre-frail	Sedentary	3.82	1.85–7.85	**<0.001**			
	Moderately active	1.36	0.57–3.23	0.485			
	Active	1					
Frail	Sedentary	12.04	4.38–33.07	**<0.001**			
	Moderately active	2.18	0.61–7.74	0.23			
	Active	1					
	Edentulism						
Pre-frail	Yes	1.475	0.74–2.92	0.266			
	No	1					
Frail	Yes	3.08	1.40–6.76	**0.005**			
	No	1					

Source: Prepared by the authors, 2025. The significance value adopted was *p* < 0.05, multinomial logistic regression; significant values highlighted in bold type. Note: Brazilian minimum salary (MS) = BRL 1518.00 (June 2025). In some items, the percentage does not reach 100% because there was data loss in “Body Mass Index” (1), “Living With Whom” (3), “Family Functionality” (10), “Chewing While Eating” (38), “High Blood Pressure”, “Hypercholesterolemia” and “Diabetes” (1), and “Edentulism” (38). Legend: EF = Complete elementary school education, MS = Minimum salary, PHCU = Primary Health Care Unit, OR: Odds ratio, CI 95% = 95% confidence interval.

**Table 4:** Factors associated with frailty in older adults according to the Functional Physical Vulnerability Index (IVCF-20), SP, Brazil, 2025.

		Functional Physical Vulnerability (IVCF-20)				
	Variables	Robust *n* (%)	PF *n* (%)	Frail *n* (%)	Total *n* (%)	*p*
Age	60 to 69	61 (57.00)	26 (35.60)	8 (20.50)	95 (43.40)	**<0.001**
	70 to 79	43 (42.20)	28 (38.40)	15 (38.50)	86 (39.30)	
	80 to 99	3 (2.80)	19 (26.00)	16 (41.00)	38 (17.40)	
Sex	Male	39 (36.40)	29 (39.70)	9 (23.10)	77 (35.20)	0.198
	Female	68 (63.60)	44 (60.30)	30 (76.90)	142 (64.80)	
Color/Race	White	68 (63.60)	45 (61.60)	27 (69.20)	140 (63.90)	0.843
	Black	37 (34.60)	25 (34.20)	11 (28.20)	73 (33.30)	
	Asian	2 (1.90)	3 (4.10)	1 (2.60)	6 (2.70)	
Body Mass Index	Obesity	21 (19.60)	16 (22.20)	11 (28.20)	48 (22.00)	0.202
	Overweight	48 (44.90)	26 (36.10)	9 (23.10)	83 (38.10)	
	Up to normal weight	38 (35.50)	30 (41.70)	19 (48.70)	87 (39.90)	
Education	Up to incomplete elementary school	30 (28.00)	32 (43.80)	30 (76.90)	92 (42.00)	**<0.001**
	EF	33 (30.80)	25 (34.20)	4 (10.30)	62 (28.30)	
	High school and above	44 (41.10)	16 (21.90)	5 (12.80)	65 (29.70)	
Marital Status	Single	50 (46.70)	37 (50.70)	28 (71.80)	115 (52.50)	**0.025**
	Married	57 (53.30)	36 (49.30)	11 (28.20)	104 (47.50)	
Living With Whom	Alone	20 (19.00)	23 (31.90)	13 (33.30)	56 (25.90)	0.080
	Spouse, family member or other	85 (81.00)	49 (68.10)	26 (66.70)	160 (74.10)	
Family Functionality (APGAR)	Severe dysfunction	13 (12.70)	11 (15.90)	16 (42.10)	40 (19.10)	**0.003**
	Moderate dysfunction	24 (23.50)	17 (24.60)	7 (18.40)	48 (23.00)	
	Good functionality	65 (63.70)	41 (59.40)	15 (39.50)	121 (57.90)	
Family Income	Up to 1 MS	38 (35.50)	37 (50.70)	25 (64.10)	100 (45.70)	**0.030**
	2 to 3 MS	50 (46.70)	27 (37.00)	11 (28.20)	88 (40.20)	
	4 MS or more	19 (17.80)	9 (12.30)	3 (7.70)	31 (14.20)	
Health Literacy	Low	36 (33.60)	40 (54.80)	32 (82.10)	108 (49.30)	**<0.001**
	High	71 (66.40)	33 (45.20)	7 (17.90)	111 (50.70)	
Attendance at Neighborhood PHCU	No	36 (33.60)	17 (23.30)	7 (17.90)	60 (27.40)	0.107
	Yes	71 (66.40)	56 (76.70)	32 (82.10)	159 (72.60)	
Physical Activity	Sedentary	44 (41.10)	41 (56.20)	29 (74.40)	114 (52.10)	**0.003**
	Moderately active	19 (17.80)	14 (19.20)	5 (12.80)	38 (17.40)	
	Active	44 (41.10)	18 (24.70)	5 (12.80)	67 (30.60)	
Chewing While Eating	Poor	4 (4.30)	6 (10.50)	3 (9.40)	13 (7.20)	0.302
	Average	22 (23.90)	19 (33.30)	10 (31.20)	51 (28.20)	
	Good	66 (71.70)	32 (56.10)	19 (59.40)	117 (64.60)	
Medications Used	3 or more per day	52 (48.60)	55 (75.30)	33 (84.60)	140 (63.90)	**<0.001**
	1 to 2 per day	47 (43.90)	17 (23.30)	5 (12.80)	69 (31.50)	
	None	8 (7.50)	1 (1.40)	1 (2.60)	10 (4.60)	
High Blood Pressure	Yes	81 (75.70)	60 (82.20)	32 (84.20)	173 (79.40)	0.411
	No	26 (24.30)	13 (17.80)	6 (15.80)	45 (20.60)	
Diabetes	Yes	38 (35.50)	40 (54.80)	21 (55.30)	99 (45.40)	**0.016**
	No	69 (64.50)	33 (45.20)	17 (44.70)	119 (54.60)	
Hypercholesterolemia	Yes	34 (31.80)	23 (31.50)	16 (42.10)	73 (33.50)	0.464
	No	73 (68.20)	50 (68.50)	22 (57.90)	145 (65.50)	
Alcoholism	Currently	18 (16.80)	3 (4.10)	6 (15.40)	27 (12.30)	0.101
	Formerly	19 (17.80)	18 (24.70)	6 (15.40)	43 (19.60)	
	Never	70 (65.40)	52 (71.20)	27 (69.20)	149 (68.00)	
Charlson Comorbidity	High and moderate risk	8 (7.50)	14 (19.2)	13 (33.3)	35 (16.00)	**0.001**
	Slight risk	99 (92.50)	59 (80.8)	26 (66.70)	184 (84.00)	
IADL (Lawton)	Partially dependent	2 (1.90)	7 (9.60)	26 (66.70)	35 (16.00)	**<0.001**
	Independent	105 (98.10)	66 (90.40)	13 (33.30)	184 (84.00)	
Depression (EGD15)	Serious depression	1 (0.90)	6 (8.20)	1 (2.60)	8 (3.70)	**<0.001**
	Mild to moderate depression	22 (20.60)	32 (43.80)	25 (64.10)	79 (36.10)	
	Normal	84 (78.50)	35 (47.90)	13 (33.30)	132 (60.30)	
Cognition (10CS)	Probable cognitive impairment	3 (2.80)	3 (4.10)	10 (25.60)	16 (7.30)	**<0.001**
	Possible cognitive impairment	20 (18.70)	25 (34.20)	16 (41.00)	61 (27.90)	
	Normal	84 (78.50)	45 (61.60)	13 (33.30)	142 (64.80)	
Edentulism	Yes	34 (37.40)	27 (47.40)	23 (69.70)	84 (46.40)	**0.006**
	No	57 (62.60)	30 (52.60)	10 (30.30)	97 (53.60)	
Total		107	73	39	219	

Source: Prepared by the authors, 2025. The significance value adopted was *p* < 0.05, chi-square test; significant values are highlighted in bold type. Note: Brazilian minimum salary (MS) = BRL 1518.00 (June 2025). In some items, the percentage does not reach 100% because there was data loss in “Body Mass Index” (1), “Living With Whom” (3), “Family Functionality” (10), “Chewing While Eating” (38), “High Blood Pressure”, “Hypercholesterolemia” and “Diabetes” (1), and “Edentulism” (38). Legend: PF = Potentially frail, EF = Complete elementary school education, MS = Minimum salary, IADL = Instrumental Activities of Daily Living, PHCU = Primary Health Care Unit.

**Table 5:** Factors associated with potential frailty and frailty in older adults through logistic regression analysis in accordance with the Functional Physical Vulnerability Index (IVCF-20), São Paulo, Brazil, 2025.

		Potentially Frail (PF) and Frail (Versus Robust)					
	Variable	Univariate Model			Multivariate Model		
		Crude OR	CI 95%	*p*	Adjusted OR	CI 95%	*p*
	Health Literacy						
PF	Low	2.39	1.30–4.40	**0.005**	1.81	0.79–4.10	0.158
	High	1			1		
Frail	Low	9.02	3.63–22.42	**<0.001**	5.65	1.83–17.44	**0.003**
	High	1			1		
	Sex						
PF	Male	1.15	0.62–2.12	0.656			
	Female	1					
Frail	Male	0.52	0.23–1.21	0.132			
	Female	1					
	Education						
PF	Up to incomplete elementary school	2.93	1.37–6.26	**0.005**			
	EF	2.08	0.96–4.51	0.063			
	High school and above	1					
Frail	Up to incomplete elementary school	8.8	3.07–25.26	**<0.001**			
	EF	1.07	0.27–4.28	0.927			
	High school and above	1					
	Marital Status						
PF	Single	1.17	0.65–2.13	0.602	1.56	0.58–4.19	0.378
	Married	1			1		
Frail	Single	2.90	1.31–6.42	**0.009**	0.35	0.09–1.27	0.109
	Married	1			1		
	Age						
PF	80 to 99	14.86	4.04–54.59	**<0.001**	28.67	5.31–154.89	**<0.001**
	70 to 79	1.53	0.79–2.96	0.209	1.35	0.56–3.25	0.509
	60 to 69	1			1		
Frail	80 to 99	40.67	9.67–171.04	**<0.001**	61.32	8.52–441.49	**<0.001**
	70 to 79	2.66	1.04–6.83	**0.042**	2.36	0.64–8.73	0.200
	60 to 69	1			1		
	Living With Whom						
PF	Alone	1.99	1.00–4.00	0.051	4.47	1.43–13.95	**0.010**
	Spouse, family member or other	1			1		
Frail	Alone	2.12	0.93–4.85	0.073	1.85	0.48–7.18	0.371
	Spouse, family member or other	1			1		
	Family Income						
PF	Up to 1 MS	2.06	0.82–5.12	0.122			
	2 to 3 MS	1.14	0.45–2.86	0.780			
	4 or more MS	1					
Frail	Up to 1 MS	4.17	1.11–15.57	**0.034**			
	2 to 3 MS	1.39	0.35–5.55	0.638			
	4 or more MS	1					
	Alcoholism						
PF	Currently	4.46	1.25–15.93	**0.021**			
	Formerly	5.68	1.42–22.64	**0.014**			
	Never	1					
Frail	Currently	1.16	0.41–3.22	0.780			
	Formerly	0.95	0.29–3.48	0.935			
	Never	1					
	Medications						
PF	3 or more medications per day	8.46	1.02–70.01	**0.048**			
	1 to 2 medications per day	2.89	0.34–24.88	0.333			
	No continuous medications	1					
Frail	3 or more medications per day	5.08	0.61–42.47	0.134			
	1 to 2 medications per day	0.85	0.09–8.27	0.889			
	No continuous medications	1					
	Diabetes						
PF	Yes	2.20	1.20–4.04	**0.011**	2.86	1.27–6.45	**0.011**
	No	1			1		
Frail	Yes	2.24	1.06–4.76	**0.035**	2.98	1.02–8.73	**0.046**
	No	1			1		
	Body Mass Index						
PF	Obesity	0.96	0.43–2.16	0.931			
	Overweight	0.69	0.35–1.35	0.275			
	Up to normal weight	1					
Frail	Obesity	1.05	0.42–2.61	0.921			
	Overweight	0.37	0.15–0.92	**0.033**			
	Up to normal weight	1					
	Attendance at Neighborhood PHCU	0.60	0.31–1.18	0.136			
PF	No	1					
	Yes	0.43	0.17–1.07	0.070			
Frail	No	1					
	Yes	0.60	0.31–1.18	0.136			
	Charlson Comorbidity Index						
PF	Moderate to very high	2.99	1.18–7.55	**0.021**			
	Mild	1					
Frail	Moderate to very high	6.19	2.32–16.50	**<0.001**			
	Mild	1					
	Family Functionality (APGAR)						
PF	Severe family dysfunction	1.22	0.49–3.04	0.670	1.12	0.34–3.67	0.855
	Moderate family dysfunction	1.19	0.58–2.46	0.640	1.14	0.44–2.99	0.778
	Good family functionality	1			1		
Frail	Severe family dysfunction	5.33	2.12–13.41	**<0.001**	6.81	1.72–27.06	**0.006**
	Moderate family dysfunction	1.26	0.46–3.48	0.650	1.93	0.51–7.32	0.332
	Good family functionality	1			1		
	Depression (EGD15)						
PF	Serious depression	14.4	1.67–124.04	0.015			
	Mild to moderate depression	3.49	1.78–6.83	<0.001			
	Normal	1					
Frail	Serious depression	6.46	0.38–109.79	0.197			
	Mild to moderate depression	7.34	3.24–16.64	**<0.001**			
	Normal	1					
	Instrumental Activities of Daily Living (Lawton)						
PF	Partially dependent	5.57	1.12–27.62	**0.036**			
	Independent	1					
Frail	Partially dependent	105	22.3–494.40	**<0.001**			
	Independent	1					
	Cognition						
PF	Probable impairment	1.87	0.36–9.63	0.456			
	Possible impairment	2.33	1.17–4.65	**0.016**			
	Normal	1					
Frail	Probable impairment	21.54	5.23–88.78	**<0.001**			
	Possible impairment	5.17	2.14–12.45	**<0.001**			
	Normal	1					
	Physical Activity						
PF	Sedentary	2.28	1.14–4.56	**0.020**			
	Moderately active	1.80	0.75–4.35	0.191			
	Active	1					
Frail	Sedentary	5.80	2.06–16.36	**0.001**			
	Moderately active	2.32	0.60–8.94	0.223			
	Active	1					
	Edentulism						
PF	Yes	1.51	0.77–2.95	0.230	1.09	0.47–2.53	0.841
	No	1			1		
Frail	Yes	3.86	1.64–9.07	**0.002**	2.08	0.66–6.55	0.211
	No	1			1		

Source: Prepared by the authors, 2025. The significance value adopted was *p* < 0.05, multinomial logistic regression; significant values highlighted in bold type. Note: Brazilian minimum salary (MS) = BRL 1518.00 (June 2025). In some items, the percentage does not reach 100% because there was data loss in “Body Mass Index”(1), “Living With Whom” (3), “Family Functionality” (10), “Chewing While Eating” (38), “High Blood Pressure”, “Hypercholesterolemia” and “Diabetes” (1), and “Edentulism” (38). Legend: PF = Potentially frail, EF = Complete elementary school education, MS = Minimum salary, PHCU = Primary Health Care Unit, OR: Odds ratio, CI 95% = 95% confidence interval.

## Data Availability

These data are available in a repository. Link: https://osf.io/jdgh7/overview?view_only=5a7bc0b29e9e44a096f84fe1e75767e0 (acessed on 22 May 2026).

## Abbreviations

The following abbreviations are used in this manuscript:

HL	Health Literacy
PHCU	Primary Health Care Unit
FHU	Family Health Unit
IADL	Instrumental Activities of Daily Living
